# Dental Hints Department

**Published:** 1906-01

**Authors:** B. H. Teague

**Affiliations:** Aiken, S. C.


### DENTAL HINTS DEPARTMENT.

Conducted by B. H. Teague, D. D. S., Aiken, S. C., to whom all contributions to this department should be sent.

Lubricating Mixture.—Castor Oil, Glycerine and Absolute Alcohol make a good lubricator. It will not gum.—*Dr. J. F. Steele, Eagle Grove, Iowa.*

When to Regulate.—It is asked: "What is really the best age to regulate, after eruption?" Just as soon as the crown can be gotten hold of to put an apparatus on; the age, of course, differs slightly with different individuals.—*E. A. Bogue, Brit. Dent. Jour.*

Dead Beat List.—St. Louis physicians and dentists have made up a black list, and no pay, no cure will be their motto in future.—*American Dental Journal.*

To Make Metal Separating Disks.—Take a sheet of copper No. 30 guage, sprinkle carbo powder over it. Place another sheet over it and run the two together through the rolling mill, separate and cut out disks from the sheets with a disk or wad cutter.—*Dr. J. F. Steele, Eagle Grove, Iowa.*

Ptyalism.—

| | |
|---|---|
| ℞—Tinct. myrrhæ, | ʒiij; |
| Potass. chloratis, | ʒss; |
| Sodii chloridi, | ℥ij; |
| Aquæ dist., | q. s. ad ℥viij. M. |

Sig.—Use as a mouth-wash. Repeat every two hours.

*Medical Press.*

To abort a felon apply a tight-fitting rubber nipple over the end of the finger as long as can be borne.—*Dr. H. Whisler, Ark. Clinic.*

Mixing Cement.—Strongly alkaline oral secretions may dissolve away the best cements, or fermenting products may also disintegrate them, but from comparing results from the various methods of manipulating cements, we believe that the average dentist does not mix his cement properly.— *Dr. N. S. Hoff, Register.*

Shock is the chief cause of death in anesthesia; when just going under or just coming out from it.—*Dr. Brunton, Ark. Clinic.*

Supporting a Sore Tooth While Drilling.—Instead of supporting a tooth by ligature to prevent pain while it is being drilled, take modeling compound, soften it and make a splint for both lingual and buccal sides of the teeth to support the sore tooth while drilling. This will prevent jarring and also prevent pressure on the inflamed peridental membrane.—*Dr. T. L. Gilmer, Review.*

Of ten severe frontal sinus suppurations treated without external operation but one was cured.—*Dr. Casselbury, Alk. Clinic.*

Another Dental Congress.—There is to be an exposition held at Jamestown, Va., in 1907, and a committee has been appointed by the Virginia State Dental Society to confer with the National Dental Society to organize a dental congress.

Missouri forbids sale of cocaine except on written prescriptions of doctor or dentist; also refilling such orders.—*Ark. Clinic.*

Spittoons in Public Places at Buenos Ayres.—The regulations at Buenos Ayres call for spittoons mounted on standards or fastened in the wall at a certain height from the floor, in all schools, colleges, theaters or other public places, the number proportional to the attendance. They must each contain water or some disinfectant. Above every cuspidor is a placard with the words: "Hygienic cuspidor. For reasons of public health it is forbidden to spit on the floor."—*Digest.*

Cracks in the Enamel.—After inserting, as we thought, a perfect gold filling, a suspicious-looking crack may suddenly appear in the enamel. This is due to the expansion of the gold in response to the heat engendered in polishing the filling; in other words, the expanding plug of gold has burst the unyielding walls of the cavity, and so surely as the gold will contract again on cooling, so surely must it leave a space between itself and the walls of the cavity.—*C. E. Brown, Record.*

EXTRACTION FOR THE CURE OF ABSCESS.—"What is our business in life? To take out an inefficient organ which is suffering for a moment with a passing disease? An abscessed tooth is very easily curable; then why should we extract it? The extraction of teeth, it seems to me, is the practice of the inefficient man. The curing of an abscess is an extremely simple thing in almost every case and the preservation of an abscessed tooth is the simple duty of the accomplished dentist. —*Dr. N. S. Jenkins, Items.*

GRINDING THE TEETH TO PRODUCE ESTHETIC LINES.—Good results may be obtained by grinding the teeth to restore the esthetic lines of the mouth. Certain lines will give the teeth an aristocratic look, and dentists should cultivate the art of fiinding the correct shape for the teeth. A line slightly one way or the other will have a great effect on the general appearance.—*Dr. H. L. Schaffner, Review.*

THE MEISTER MATRIX is a very useful instrument. After an amalgam filling is inserted with it which requires an all-around matrix—the ends of the matrix can be separated from the clamp or holder and then tied with a strong ligature and allowed to remain until the following day, when it is removed and the filling shaped and polished.—*Wes. D. Jour.*

THE BROACH.—As I never use any other than a soft broach, I find the most convenient and effective method of sterilization is to pass the broach through the flame of the alcohol lamp just before using it. It should be held a little above the flame, to avoid burning it, and should not be withdrawn from the heat too quickly, or the sudden cooling will harden it.—*Dr. J. Leon Williams. Dental Cosmos.*

PULP MUMMIFICATION.—I never mummify a pulp that comes to me in any other condition than freshly exposed. Some try to mummify pulps that are diseased, pulps with pulpstones, teeth that are loose in the socket. That is where the failures come in. The pulp must be in a comparatively healthy condition.—*Dr. Waas, Items of Interest.*

LOCAL ANESTHESIA FOR EXTRACTION.—The pain of extraction is caused by the tearing of the alveolo-dental ligament; therefore if anesthesia by cocain is to be effective it must act on the nerve termini of this ligament. To insure the efficacy of the injection it must be made at the level of the mucous membrane, which adheres closely to the periosteum and consequently not too near the neck.—*Dr. E. Sauvez, Brit. Dent. Journal.*

Combination Fillings; Gold and Tin.—If three sheets of gold, No. 6 or 8, and one of tin are folded in three, cut into strips and packed into a cavity, not only crowns but interstitial cavities in bicuspids can be satisfactorily filled, and such fillinsg have been found to last in mouths where other fillings have not proved a success.—*Digest.*

Anesthetic Crazed Patient.—This month a man at Sag Harbor, L. I., while under the effects of an anesthetic given prior to the extraction of some teeth, suddenly became violent, kicked out the window in front of the dental chair, and drove the dentist and his assistant from the office. He soon recovered his senses, but the dentist declined to do any further work.—*Digest.*

Ancient Remedy for Baldness.—"A mixture of dogs' paws, dates, and asses' hoofs, ground up and cooked in oil. The head to be rubbed vigorously with the preparation."—*Good Health.*

To Collect Spilled Mercury.—If mercury is accidentally spilled on table or floor, make a wet ring around it; it will be found that the globules of mercury cannot readily cross this ring, and the mercury can be collected without difficulty in a small shovel made from a piece of thin card or even in an ordinary envelope.—*British Dental Journal.*

Sterilization of Cutting Instruments.—Immersion in 95 per cent. alcohol has the least effect in dulling the edge of a knife; boiling has the most effect.—*Western Dental Journal.*

Modelling Composition.—In spite of a rather general feeling to the contrary the successful handling of composition requires quite as much skill as does plaster. The greater one's experience with it, the more positive becomes the opinion that the best results are to be obtained, when the least thickness possible of the softened material is used.—*S. E. Davenport, International Dental Journal.*

To Harden and Protect Plaster Casts.—Immerse the casts in a pot of boiling beeswax until thoroughly saturated. The wax penetrates throughout the paster, making it hard or horny and impervious to moisture.—*International Dental Journal.*

Pulp Devitalization.—My method is to apply arsenic fiber to the exposed pulp, if not inflamed, for from twenty-four to forty-eight hours, and then under aseptic precautions to remove the bulbous portion of the pulp and place in contact with the stump a 5 per cent. solution of formalin. This is left in contact sealed in for three days, when we find the pulp of the consistence of catgut and readily removed with the pliers.—*J. I. Hart, Dental Cosmos.*

An Old Fashioned Recipe for Tooth Powder.—"Take of Jesuit's bark, one ounce; myrrh, one ounce; orris-root powder, half an ounce; coral powder, half an ounce calcined; oyster shells, quarter of an ounce. Let the ingredients be well mixed together; dry, and they are immediately fit for use."—*Ladies' Magazine* (1803), *Brief.*

Dressing for Pulpless Teeth.—Creasoform, a light green powder, without smell or taste, soluble in alcohol and ether, and which decomposes in contact with animal tissues into creosote and nascent formaldehyd, is useful as a dressing for pulpless teeth; make into a paste with any of the essential oils.—*B. J. T. Bennette, Dental Record.*

Taking Impressions.—In cases that are extremely sensitive and easily nauseated, I have found it useful and helpful to sponge the mouth with hydrogen dioxid, then applying a 3-100 solution of eucain to the whole palate.—*T. B. Hartzell, Texas Dental Journal.*

The Soldering of Aluminum.—Herr Wagner of Berlin recommends the following for soldering aluminum and its alloys: He uses a flux formed of 80 parts stearic acid, 10 parts chlorid of zinc, 10 parts chlorid of tin. Any ordinary soft solder can be employed, but the best is that composed of 80 parts tin and 20 parts zinc. After the dentures to be soldered have been cleansed and passed through the flux the soldering is done in the usual way.—*Z., Quarterly Circular.*

Alcohol and the Teeth.—It has been noticed that spirit and beer drinkers who take small quantities of spirits, sipping them over long periods of time have defective teeth and gums, while others who drink spirits with large quantities of water, swallowing them rapidly, do not suffer so much. Persons who use beer and spirits to gratify the sense of taste, and enjoy the flavor always suffer from degeneration of the mucus membrane, gums and teeth.—*T. D. Crothers, M. D., International Dental Journal.*

Quickly Made Gold Inlay.—The following method of making gold inlay in the occlusal surface of a molar was demonstrated by Dr. W. A. Fillman. After adjusting a good matrix he packed it full of moss fiber gold and removing the mass in the matrix flowed solder into the gold. He then replaced this work in the cavity readapted to the margins with burnishers and added more solder to proper contour. With care an investment is unnecessary.—*Northwestern Dental Journal.*

Burning from X Rays.—Mr. H. Lyle, Senior Surgeon to the Liverpool Hospital for Cancer and Skin Diseases, in a letter to the *Lancet,* states that burns caused by the X rays, which are generally found to be so intractable, are readily cured by the following ointment, the dermatitis soon disappearing:

| | |
|---|---|
| ℞—Plumb. oxid., | ʒj; |
| Zinc. carb., | ʒij; |
| Glycerin, | ʒj; |
| Ol. oliv., | ʒss; |
| Adeps benzoat., | ad ʒj. |
| M. ft. ung. | |

Sig.—To be applied freely.

*Digest.*

The Oral Secretions.—I find in my practice that in a patient whose oral secretions are thick and ropy and not changed rapidly, there is a much greater accumulation of lime deposits about the neck of the teeth, and after these deposits are removed, there is a rapid recurrence of them so that removal of the deposits alone will not cure the condition. A change of environment or a change of the mode of living, together with systematic medication, bringing about a more healthy condition of the general system, may effect a cure for a time, or until similar conditons are again brought about.—*J. A. Freeman, Dental Review.*

ANTIDOTE FOR CARBOLIC ACID POISONING.—Some time ago a veterinary surgeon of Dublin accidentally discovered that turpentine, when taken in poisonous doses, is an antidote to carbolic acid. He was called upon to treat two horses that had been poisoned, and gave one of them what he thought was olive oil, but which was really turpentine. The benefit was so great that he gave some to the second horse. Both recovered rapidly, although the second horse was alreadv unconscious when the dose was administered. As we have at the present time no very efficacious treatment for this form of poisoning—which is a favorite method of many would-be suicides—it might be well worth trying to prove its value.—*Medical Age.*

MEDICAL ADVICE BY TELEPHONE.—Husband (to Physician)—My wife has a severe pain in the back of her neck, and complains of a sort of soreness in the stomach.

Physician (to Husband)—She has malaria colic.

Husband (to Physician)—What shall I do for her?

Girl at Central switches off to a machinist talking to a saw-mill man.

Machinist (to Husband)—I think she is covered with scales about one inch thick. Draw off her water and let her cool off during the night. Before she fires up in the morning clean out her flue and pound her thoroughly; then take a hose, hitch it to a fire-plug, and wash her out. Do not crowd her too much, as she is liable to blow off.

Husband has no further use for this doctor.—*Med. Chir. Jour.*

THE IDEAL DIET.—Prof. W. O. Atwater, special agent of the United States government, in charge of the nutrition investigations in various experiment stations maintained by the United States Department of Agrculture, closes one of his most interesting and valuable reports—Farmers' Bulletin No. 142—with the following words:—

"It should always be remembered that 'the ideal diet is that combination of foods which, while imposing the least burden on the body, supplies it with exactly sufficient materal to meet its wants,' and that any disregard of such a standard must inevitably prevent the best development of our powers."—*Good Health.*

PERNICIOUS WASTE.—A well-educated Englishman, I mean one well-educated in general subjects, would wonder beyond measure if he realized the enormous amount of work an Indian can do on a mere handful of rice and a few dates. But his wonder would be far more increased if, in the physiological laboratory, he were shown and made to understand three facts: (1) The exceedingly small amount of flesh-forming matter that is called for to make up the waste of the muscular organs; (2) The enormous amount of wasted material that is thrown off or laid by without having been applied to any useful purpose in the body; (3) the tremendous measure of living energy that has been expended in throwing off from the body substances which ought never to have been put into it.—*Sir Benjamin Ward Richardson, Good Health.*

My use of the 10 per cent solution of formaldehyde, if you remember, was in those cases of dead pulps where there was some approach toward putrescence, if not actual putrescence, and the object is, as I said, twofold. In the first place, it tends to dry up the pulp tissue and make it more easily manipulated, when you remove it by instrumentation; secondly, in order to make the pulp cavity as sterile as possible before instrumentation, and to avoid if possible the danger of forcing some infected matter through the end of the root. I do not claim that by flooding a canal which is filled with a putrescent mass in a liquid state, that you can avoid the infection to the end of the root; I do not claim that, though in some of those cases, but where the pulp has not reached that stage, I am sure that the use of the solution is of great advantage in preventing an infection beyond where the infection has already reached.—*Dr. M. L. Rhein, Discussion-Gazette.*

*Germs.*—In these days of scientific discovery we fear too exclusive attention is being paid to germs. Almost every human malady has been traced directly or indirectly to micro-organisms. There is a tendency to overlook the great fundamental fact that germs are, after all, only the exciting cause of disease. The healthy organism is thoroughly able to defend itself against all comers in the shape of germs. Bad habits open the door to the germ enemies of life by producing conditons which favor infection and germ growth within the body. Wrong habits of eating and drinking, neglect of exercise and other matters essential to health, break down the body and leave it an easy prey to scavenger and parasitic organisms, which are powerless to invade a healthy body.—*Good Health.*

Talking by Mail.—Now comes the phonopostal, which records and reproduces the voice on a postal-card-shaped piece of pasteboard. A common phonograph makes the record with a sapphire-pointed stylus. This graves deeply an easily spread, impressionable material, called "sonorine," on the surface of the card. The sounds are written spirally, beginning at the edge and continuing in a narrow curve whch ends in a little circle no bigger than a ten-cent piece. "Sonorine" is healthy enough to survive rough usage in the mails. A "phonocard"—how quickly language grows in these days!—will hold, say, eighty words. It is expected to take the place of the illustrated postal card, which is becoming too ancient for this highly modern world.—*"With the Procession," Everybody's Magazine for December.*

